# Supplementary material for: Are carbon emissions trading and green financial instruments synergistic? -Comprehensive quantitative research based on content analysis
Source: PLoS One. 2024 Mar 7;19(3):e0298601. doi: 10.1371/journal.pone.0298601 (PMC10919601; doi:10.1371/journal.pone.0298601)
Supplement: S1 Data — (ZIP) [file pone.0298601.s001.zip › Data/地级市Z_F工作报告.zip!/╔╜╢1⁄2/┴┘╥╩╩╨/└┤╫╘╣1⁄2╓┌║┼í╛┬φ┐╦╩2╛▌═°í┐_2016.docx]

各位代表：

现在，我代表市人民政府向大会作政府工作报告，请予审议，并请市政协委员和其他列席会议的同志提出意见。

一、“十二五”规划完成情况及2015年工作回顾

2011年至2015年，是临沂深化改革、扩大开放、加快发展的五年。五年来，在市委的坚强领导下，全市上下认真贯彻落实中央和省委、省政府一系列决策部署，牢牢把握稳中求进总基调，沉着应对挑战，科学谋划发展，统筹做好各项工作，顺利完成了“十二五”规划确定的主要目标任务。全市地区生产总值达到 3763.2亿元，年均增长10.4%;一般公共预算收入283.9亿元，是2010年的2.5倍;固定资产投资、社会消费品零售总额、进出口总额年均分别增长19.4%、14%和12.8%，主要经济指标增幅居全省前列。

回首“十二五”，发展有目共睹，成绩可圈可点：

——主导产业在“调整优化”中提档升级。三次产业比例由2010年的11：50.3：38.7调整到9.2：44.8：46。工业总产值突破万亿元，比“十一五”末翻了一番，成为全省第6个、全国第29个工业产值过万亿的地级市。主导产业成长快、前景好。医药产业，在鲁南制药、罗欣药业等骨干企业的带动下，制药企业发展到近百家，主营业务收入、利税分别增长2倍和1.5倍;装备制造业，以临工、常林为代表的600多家企业，技术水平和市场竞争力不断提升，主营业务收入和利税均实现翻番;食品产业，以金锣、鲁洲为骨干的近700家规模以上企业，主营业务收入突破2000亿元，成为工业第一大产业;新能源、新材料、信息技术等战略性新兴产业从无到有、从小到大，都已成长为百亿级产业。新能源汽车和整装汽车制造产能分别达到10万辆、30万辆。高新技术产业产值占比提高6个百分点。万元工业增加值能耗年均下降12%，万元GDP能耗完成省政府下达任务。临沂商城市场交易额年均增长21%，物流业增加值占比保持全省首位。存贷款余额分别达到4762亿元和3400亿元，全国性股份银行、城市商业银行发展到13家，场外市场挂牌企业77家。文化产业增加值占比超过5%，累计接待游客2亿多人次，蒙山旅游区被评为国家5A级景区。粮食综合生产能力稳步提高，认证“三品一标”产品1737个、数量居全省第一。畜牧业、渔业产值分别增长31%和55.6%，农机化水平达到85.5%。河东农高区创建成为国家级农业科技园区。

——城乡建设在“四级联动”中协调推进。全市城镇化率达到55.3%，年均提高1.5个百分点。中心城区人口和建成区面积分别达到205万人、213 平方公里。沂水县被授予全国文明县城称号，沂南、莒南、临沭、兰陵和费县等成为国家园林县城。乡镇新一轮总体规划修编基本完成，19个小城镇被确定为全国重点镇，22个被列为省级示范镇，12个农村新型社区入选全省示范社区。临枣、长深高速和蒙山高架路、环城快速路等重点交通工程建成通车，临沂机场航空口岸获准开放。30万吨供水东线工程和西部供热中心等投入运营。完成大型病险水库和塘坝除险加固276座，治理中小河流265公里，在全省率先建成数字信息化防汛指挥体系，获批全国水生态文明建设试点城市。成功创建全国文明城市和国家卫生城市、森林城市、环保模范城市，实现全国双拥模范城“三连冠”。先后被评为中国物流之都、板材之都和全国科技进步先进市、首批生态文明先行示范区。

——改革开放在“内强外拓”中释放活力。简政放权力度加大，共取消、下放市级行政审批事项277项，清理削减行政权力事项1927项。商事制度改革深入实施，市场主体登记实现“三证合一、一照一码”，“个转企、小升规、规改股、股上市”取得积极成效。财税金融改革有序开展，“创新联银合作新组织破解小微企业融资难题”的做法，荣获全国工商联系统“创新中国特别奖”。新一轮政府机构改革基本完成，事业单位分类改革稳妥推进。农村产权制度、县域金融创新等 17项国家级和16项省级改革试点进展顺利。科技创新日益活跃，建成国家级创新平台18家、省级115家，荣获国家科技进步奖6项，新时代药业获中国专利金奖。“走出去”“请进来”步伐加快，累计引进市外资金近3000亿元，全市境外企业超过100家，有权出口企业近5000家，临工、立晨等一批境外合作项目建设顺利，绿润、山松等20多家企业实现境外上市。高新区和经开区升格为国家级开发区，临港区引进全国首家“互联网+”、“文化+”双加实验室，各类平台成为对外开放和创新发展新高地。

——公共服务在“民生优先”中更加均衡。民生支出年均增长18.6%，占财政支出比重保持79%以上。城镇和农村居民人均可支配收入年均增长 11.8%、13.4%。新增城镇就业68万人，农村劳动力转移就业118万人，在全国率先启用农民工“一元公寓”，市技师学院被确定为国家级高技能人才培训基地。连年提高企业退休人员基本养老金。11个县区通过国家县域义务教育均衡发展验收，所有县区都有1家以上医院达到二级甲等水平。建设保障性安居工程16.6万套，310万农村居民饮水安全问题基本得到解决。沂蒙革命纪念馆落成开馆，柳琴戏《沂蒙情》获“五个一”精品工程奖，市图书馆、科技馆、文化中心、临沂大剧院、东夷文化园等成为群众休闲娱乐好去处，城乡居民的幸福感不断增强。

各位代表，刚刚过去的2015年，是“十二五”规划的收官之年。面对宏观经济下行压力，全市上下按照“走在前列”要求，认真落实市委十二届八次、九次、十次全会精神，适应经济新常态，实施“八大战略”，打好“六场硬仗”，经济发展保持了总体平稳、稳中有进的基本态势，“大美新”临沂建设迈出更加坚实步伐。

(一)“三引一促”初见成效。各级各部门立足职能抓招引、发挥优势抓招引、明确分工抓招引，初步形成了招引工作合力。紧盯产业重点区域、国内外500强企业，精心策划项目，主动对接项目，提高了招商成功率;瞄准科技发展前沿，引进创新团队和高新技术成果，提高了招商含金量。成功举办杭州招商引资洽谈会、百名外国专家沂蒙行等推介活动。全市共引建招商引资项目 1016个，到位市外资金1290.5亿元、增长119%。众泰汽车及发动机、盼盼食品、宜华木业等一批重点产业项目开工建设，中兴通讯、修正药业等一批新兴产业项目落户临沂。先后引进先进技术569项、“千人计划”专家和“泰山学者”等高层次人才225名、创新创业人才1076名。

(二)“二次创业”拉开序幕。引导企业主动适应新常态，科学制定“二次创业”计划，转变发展理念，创新发展路径，实施高点对标，企业发展动力明显增强。以大气污染防治为契机，严格执法抓环保，利用环保促转调，倒逼企业转型升级。六大高耗能行业产值下降11.6%，淘汰钢铁产能150万吨、焦化产能 260万吨，关停非煤矿山88个、30万吨以下煤矿5个，改造提升建陶生产线101条，上万家木业企业实现了统一供气或清洁能源利用。鼓励企业上项目、增投入，新增规模以上工业企业339家、总数突破4000家，产值过亿元企业2371家。实施“千企千项千亿”工程，支持企业加快技术改造，完成工业技改投资1039.8亿元、增长16.7%。全市“双百”企业实现主营业务收入3250亿元、利税220亿元，分别占规模以上工业的33%和29%。金锣、天元等4家企业入选“2015中国企业500强”，金升、金正大等6家企业入围“2015中国民营企业500强”。

(三)商城国际化加快推进。主动融入“一带一路”战略，扎实开展国际贸易综合改革试点，推进内贸与外贸、线上与线下、商贸与生产融合发展，商城国际化步伐明显加快。临沂商城市场交易额突破3200亿元，全市出口总额、物流总额、电子商务交易额分别达到60.6亿美元、2.5万亿元和1100亿元。临沂港运行良好，综合保税区开关运行。“智慧临沂商城”上线运营，“市场采购”贸易方式有望近期获批。“海外临沂商城”加快建设，匈牙利中欧商贸物流合作园区被认定为首个国家级境外商贸物流型经贸合作区。全市建成各类电商园区17处，培育电商平台30个、龙头企业60家，发展网商6万户，获评中国电子商务经营百强城市。鲁南铁路物流园被列为国家亮点工程，满易网入选国家商贸物流标准化专项行动，临沂至乌鲁木齐、昆明、西宁和“临满欧”等货运班列陆续开行。物流促商贸、商贸活经济的格局进一步形成。

(四)新型城镇化健康发展。编制完成了《临沂市新型城镇化规划》、《北城新区二期控制性详细规划》等20个规划，中心城区新一轮控规覆盖率达75%。北城新区二期、西部新城、火车站片区、汤泉旅游区、临空经济区等重点片区加快建设，东风东关片区等一批棚改项目顺利实施，鲁商中心、齐鲁园广场和政务服务中心等项目即将投入使用。实施“四供两排”和城市绿化工程，扩建城市污水处理厂5座，集中供热、管道燃气、公共供水普及率分别达到65%、91%和 70%，绿化覆盖率、绿地率分别为41%和37%。坚持统分结合、重心下移，科学划分市区城市管理职责，城乡环卫一体化实现全覆盖。推进以城带乡、联动发展，沂水县省级新型城镇化试点正式启动，兰山区义堂镇被列为国家新型城镇化试点，有13个重点镇进入全省百强。新建农村新型社区50个，农村住房和危房改造7万余户。鲁南高速铁路临沂至曲阜段、临沂机场三期工程、罗岚高速公路启动建设，沂沭铁路建成通车，北疏港公路基础工作基本完成。

(五)生态环境整治扭转被动局面。全面打响大气污染防治攻坚战，工业污染、城市扬尘、机动车尾气、秸秆禁烧等综合整治取得阶段性成果。空气质量综合指数位居全省第七，改善幅度全省首位，“沂蒙蓝”得以重现，国家环保部和省委、省政府对“临沂样本”给予充分肯定。实施中心城区6条河流综合整治，高标准建设国家规划项目78个，增建治污项目1122个，8个国控河流断面水质全部达标，代表山东省迎接国家淮河流域核查实现“八连冠”。建设生态系统和绿色屏障，新造林地35.5万亩，绿化城区裸露土地319万平方米，整治露天采矿点98个。创建全国绿化模范县2个、中国十大最美乡村2个、省级以上生态县区4 个。严把新上项目用能关，实行能耗总量和强度双控制，省政府下达的年度节能目标圆满完成。

(六)民生保障更加有力。持续抓好富民、惠民、安民工程，群众关心的热点难点问题正逐步得到解决。统筹做好重点群体就业工作，深入开展全民创业行动，新增城镇就业13.4万人，农村劳动力转移就业17.1万人。扎实推进社保提标扩面，城乡居民养老保险基础养老金每人每月提高到85元，农村低保年均保障标准提高到3031元、城市低保月均保障标准提高到416元，农村五保集中和分散供养标准分别提高到每人每年4500元、3200元，五保集中供养率达到 70%以上。全面推行县级公立医院改革，城乡居民医保参保率、受益人数位居全省前列。坚持教育优先，新建、改扩建校舍1144万平方米，县域义务教育均衡发展和“全面改薄”经验在全省推广，我市被列入全国基础教育课程综合改革示范实验区。实施精准扶贫、精准脱贫，实现减贫21.8万人。健全四级食品药品安全监管体系，创建“食安山东”品牌154个。临沂大剧院、文化广场、书法广场等文化设施运行良好。群众性精神文明创建、民主法治建设深入推进。社会治安综合治理投入不断加大，基层基础工作进一步夯实，群众安全感满意度有了新的提高。

各位代表，过去一年，我们扎实开展“三严三实”专题教育，积极参加“结亲连心”活动，持之以恒纠“四风”、转作风，着力打造有为、高效、诚信、清廉政府，各级政府现代治理能力进一步提高。深化行政审批制度改革，建立“四清单、一平台”政府权力运行体系，在全省率先完成市级行政审批事项目录调整规范。自觉接受人大法律监督、政协民主监督和社会监督，人大代表建议和政协委员提案办结率达到100%。完善四级便民服务体系，96%的市级行政审批事项进驻市政务服务中心，办理时限缩短一半以上。市民服务热线受理来电近200万件，诉求办结率达98%。认真履行党风廉政建设责任制，依法实施行政监察和审计监督，把牢法纪红线、政策底线、职责界线和安全防线，勤政廉政建设取得明显成效。国防动员、人民防空、民兵预备役工作全面加强，妇女儿童、民族宗教、老龄、对台、外事、侨务、史志、档案、保密、气象、防震减灾和红十字会等工作都有新进展。

各位代表，五年的风雨历程，谱写了沂蒙大地发展的新篇章;五年的辉煌业绩，标志着临沂已经站在新的历史起点上。五年来，我们面对挑战不退缩，抢抓机遇不懈怠，以提高质量效益为中心，在“转”中提档升级，在“调”中优化结构，在“创”中增添动力，推动经济有质量、有效益、可持续增长。五年来，我们科学谋划、奋力开拓，用改革的办法破解难题，用开放的思维配置资源，用创新的举措明晰路径，发展活力不断增强。五年来，我们坚持生态立市不动摇，实行源头严防、过程严管、后果严惩，治气治水一起抓，治标治本齐发力，积极探索绿色发展新路径，推动生态环保与经济建设互促共赢。五年来，我们始终心存民之所望、政之所向的情怀，恪守为民之责，多办利民之事，千方百计保基本、补短板、兜底线，实现了居民生活有提高、弱势群体能救助、群众权益得保障的目标。五年来，我们秉承打基础、利长远的发展理念，保持“功成不必在我”的境界，一张蓝图绘到底、一任接着一任干，求真务实、拼搏进取，以实实在在的业绩赢得群众信任和支持。

五年来的实践证明，我们经受住了复杂局面的考验，拓展了转型升级的空间，积蓄了科学发展的后劲。成绩来之不易，经验弥足珍贵。这是省委、省政府和市委正确领导的结果，是市人大、市政协监督支持的结果，是历届领导班子接力奋斗的结果，是全市人民团结实干的结果，也是各方面关心帮助的结果。在此，我代表市人民政府，向全市广大干部群众，向人大代表、政协委员、各民主党派、工商联、人民团体和离退休干部，向驻临人民解放军、武警官兵和中央、省属单位，向所有关心支持临沂发展的各界朋友，表示衷心的感谢和崇高的敬意!

在肯定成绩的同时，我们也应清醒地看到发展中存在的困难和问题：经济下行压力仍在持续，企业生产经营困难加大，金融、房地产等领域存在一定风险;传统产业层次不高，新兴产业比重较小，淘汰落后产能任务艰巨;企业创新能力不足，高层次人才和具有核心竞争力的技术匮乏;县域经济实力不强，基础设施建设相对滞后，城乡发展还不平衡;资源环境约束趋紧，大气、水、土壤等污染问题比较突出;民生保障存在不少短板，部分群众生产生活仍然困难，脱贫攻坚任务相当繁重;安全生产形势依然严峻，扭转被动局面亟需持续用力;政府职能有待进一步转变，营商环境尚需优化，等等。对这些问题，我们一定高度重视，深入研究，尽快解决。二、“十三五”时期的主要目标和重点任务

根据党的十八届五中全会、省委十届十三次全会和《中共临沂市委关于制定临沂市国民经济和社会发展第十三个五年规划的建议》精神，我们编制了《“十三五”规划纲要(草案)》。纲要(草案)及说明已印发各位代表，请予审议。

“十三五”时期是我国全面建成小康社会的决胜阶段，也是我市内强外拓、发力赶超的关键时期。当前，世界经济仍处于深度调整期，国内经济企稳回升的基础尚不稳固，需要克服的困难还很多。在增强忧患意识、强化底线思维的同时，也要看到我国经济发展长期向好的基本面没有变，经济韧性好、潜力足、回旋余地大的基本特征没有变，持续增长的良好支撑基础和条件没有变，经济结构调整优化的前进态势没有变。实现科学跨越发展，我们面临着诸多有利条件：体现在政策红利上，随着国家“一带一路”战略的推进、革命老区开发建设指导意见的实施，以及省“两区一圈一带”规划的落地，我市面临一系列新的政策机遇。只要找到结合点、找准切入点，就一定能够把政策红利转化为现实生产力。体现在改革动力上，随着全面深化改革特别是供给侧结构性改革的深入，制约转型发展的体制性机制性难题将逐步破除。只要顺应时势、大胆探索，坚决落实去产能、去库存、去杠杆、降成本、补短板“五大任务”，做到优化存量、引导增量、主动减量，就一定能够激发出科学发展的新动能。体现在开放活力上，随着基础设施的完善、立体大交通格局的形成，特别是商城国际化步伐的加快，我市对外开放面临新的机遇。只要利用好国内国际两个市场、两种资源，发挥好商城国际化的引领作用，坚持内外需协调、引进来走出去并重、引资引智并举，就一定能够拓展双向开放新空间，推动开放型经济迈向更高水平。体现在发展潜力上，随着“八大战略”、“六场硬仗”的全面铺开，以及“三引一促”、“二次创业”和新型城镇化等重大部署的深入实施，经济社会发展将踏上全面建成小康社会的新征程。只要我们准确把握重要战略机遇期内涵的深刻变化，积极作为、乘势而上，就一定能够早日实现“大美新”临沂建设的宏伟目标!

“十三五”时期，全市经济社会发展总的要求是：深入贯彻党的十八大和十八届三中、四中、五中全会以及习近平总书记系列重要讲话精神，按照“走在前列” 要求，以创新、协调、绿色、开放、共享发展理念为引领，以稳中求进为总基调，以弘扬沂蒙精神为动力，积极适应经济发展新常态，更加注重提高质量和效益，更加注重以人为本，更加注重促进形成绿色生产方式和消费方式，更加注重对特定人群特殊困难家庭的精准帮扶，更加注重推进高水平双向开放，攻坚克难、扎实工作，确保与全省同步全面建成小康社会。到2020年，全市地区生产总值达到5600亿元，年均增长8%左右。一般公共预算收入突破450亿元，年均增长 10%左右。固定资产投资、社会消费品零售总额和进出口总额年均分别增长13%、11%和8%左右。主要目标任务是：

——壮大主导产业，建设实力城市。紧跟“中国制造2025”和“互联网+”等产业新趋势，着力提升汽车及装备制造、健康食品、中药及生物医药、高端木业等产业层次，加快打造高端液压元件及系统产业集聚区、新医药产业集聚区，推进产业向智能化、高端化升级。大力发展商贸物流、金融保险、文化旅游、养老养生等现代服务业，建设“一带一路”国际商贸物流双节点城市、区域性供应链服务及金融结算中心城市。积极发展资源节约、环境友好型现代农业，促进产出高效、产品安全，整建制成为国家现代农业示范区。到2020年，全市八大主导产业产值达到1.5万亿元，高新技术产业、战略性新兴产业和现代服务业在经济结构中的比重逐年提高，临沂商城市场交易额突破万亿元。县域经济进入赶超跨越发展新阶段，全国百强县实现“零的突破”。提前实现经济总量和城乡居民收入比 2010年翻一番，综合实力和竞争力迈入全省第一方阵，在鲁南苏北地区位次前移，在全国革命老区中率先发展。

——优化城乡环境，建设宜居城市。按照“以河为轴、两岸开发、组团发展、北上东拓”的思路，加快北城新区二期及高铁片区、西部商贸物流园区建设，推动中心城区和东部临港区、莒南县、临沭县融合发展，促进周边县区协调联动。启动中心城区轨道交通前期工作，建成运营鲁南高铁临沂至曲阜段和临沂至日照段，推动京沪高铁东线规划建设，争取成为铁路枢纽城市。落实国家主体功能区规划，从源头上保护生态环境，打造国家生态文明先行示范区，努力让临沂天更蓝、地更绿、水更清。统筹城乡基础设施、产业培育、公共服务和人口布局，推动城乡一体化发展。到2020年，中心城区人口和建成区面积分别达到300万人、320 平方公里，所有县城人口规模超过20万人，全市常住人口和户籍人口城镇化率分别达到62%和50%左右。

——繁荣文化事业，建设魅力城市。深入挖掘历史文化内涵，建设与历史资源和政治优势相匹配的文化大市，打造以红色文化为城市精神的临沂文化品牌。充分发挥山东沂蒙党的群众路线教育基地功能，创建国家红色文化教育培训示范区。发挥书圣文化节等节会活动的带动效应，大力发展文化产业，彰显历史文化、红色文化、山水文化特色。加强公共文化设施建设，综合利用基层文化资源，鼓励社会力量参与文化服务，打造15-20分钟公共文化服务圈。用好临沂大剧院、图书馆、博物馆、科技馆等平台，构建覆盖城乡、发展均衡的现代公共文化服务体系，让沂蒙文化基因代代传承、文化魅力人人称颂、文化品牌口口相传。

——深化改革创新，建设活力城市。持续推进商事及行政审批制度改革，实现审批事项数量最少、速度最快、收费最低、市场主体和人民群众最满意的目标。大力推广政府与社会资本合作模式，发挥市场力量在建设基础设施、提供公共服务中的积极作用。用好产业引导基金，加强股权投资运作，放大财政资金效应，以市场化方式促进产业发展。深化农村集体经济改革，构建现代农业生产经营服务体系。加快建立现代企业制度，抓好互联网等前沿技术及新业态、新机制、新模式的宣传，提升企业家创新创业能力。加强城乡社区治理和基层自治组织建设，激发社会组织共治活力。健全社会信用体系，建立守信激励、失信惩戒联动机制。重点领域和关键环节改革取得重要成果，各方面制度更加成熟定型。

——保障改善民生，建设幸福城市。大力实施民生优先战略，全面落实民生政策，扎实做好10个方面35项民生实事，抓发展促增收、抓扶贫促脱贫、抓就业促创业、抓普惠促民享、抓监管促安全，提升群众幸福指数。到2018年，贫困家庭人均纯收入达到全市农民人均纯收入的50%以上，贫困村全部“摘帽”，农村人口全部脱贫。到2020年，全市民生投入占比高于全省平均水平、高于周边地市水平;城乡居民收入增幅超过GDP增幅，农村居民收入增幅超过城镇居民收入增幅;确保总体实现基本公共服务均等化，与全省同步、比全国提前全面建成小康社会，努力让老区人民“劳有多得、学有优教、病有良医、出有畅行、贫有善济、居有长安”，进一步夯实“民生大厦”根基。三、2016年工作安排

今年是实施“十三五”规划、全面建成小康社会决胜阶段的开局之年，是推进结构性改革的攻坚之年，也是临沂经济转型升级的关键之年，做好今年工作意义重大。综合考虑各方面因素，今年全市经济社会发展的主要目标是：地区生产总值增长8%，一般公共预算收入增长9%，固定资产投资增长13%，社会消费品零售总额增长10%，进出口总额增长6%，城镇和农村居民人均可支配收入分别增长8%和9%以上，城镇登记失业率控制在3.5%以内，全面完成省政府下达的年度节能减排约束性指标，确保空气质量排名位次继续前移。今年的政府工作，突出抓好六个方面：

(一)推动产业迈向中高端。坚持向结构调整要增长、要效益，瞄准产业链、价值链高端，新上一批具有较大带动力的优质项目，做大一批具有较强成长性的骨干企业，发展一批具有较高市场份额的主打产品，着力构建现代产业体系。实施工业转型升级行动计划，坚持“敲开核桃、一业一策”，对木业、建材、化工、冶金等传统产业，逐一制定具体转型方案。用好环保倒逼、安全生产倒逼和节能减排倒逼“三个工具”，稳妥实施“三个一批”，即坚决淘汰一批落后产能，对能耗高、污染重的企业，落实“关、并”措施，促其有序退出市场。下决心改造一批传统产业，落实优惠政策，引导企业加快“二次创业”，实施技术改造和设备更新，运用物联网、云计算、大数据等现代信息技术，提升企业自动化、智能化水平。加快培育一批新兴产业，放大产业引导基金效应，鼓励和扶持新信息、新材料、节能环保等产业做大做强。创新园区发展模式，构建精简高效、运行顺畅、服务优质的管理体制。围绕培植主导产业，积极吸引上下游企业集聚，力争每个国家级开发区至少引进两个世界500强项目，每个省级开发区至少引进两个国内100强或行业10强项目，实现园区实力在全省、全国位次前移。培育服务业新业态，引导现代金融、科技信息、文化创意、研发设计等新兴产业加快发展，启动国家全域旅游示范市创建工作，积极发展养老服务、医疗健康等产业，使服务业成为最具活力的增长点。大力发展现代农业，落实藏粮于地、藏粮于技战略，提高粮食产能，确保粮食安全。用好农村产权制度改革成果，推进适度规模经营，促进粮经饲统筹、农林牧渔结合、种养加一体、一二三产融合发展。培育壮大龙头企业、农民专业合作社、家庭农场等新型生产经营主体，提高规模化、标准化、品牌化水平。新建现代农业示范园区20个，提升优质农产品基地100万亩。扎实开展农业面源污染防治，加强质量安全监管，积极争创全国农产品质量安全放心市。继续推进“三引一促”，瞄准国内外500强，集中力量招大商、招群商，着力引进一批大项目好项目。用好应用科学城、创业大学等各类孵化器，吸引更多高端人才来临沂创新创业。积极推进金锣文瑞、金正大诺贝丰、鲁南现代中药产业园等工业项目建设，加快开发红色沂蒙旅游园区等服务业项目，重点建设马泉创意农业休闲园、兰陵现代蔬菜种业研发中心等农业项目，进一步发挥重点项目的带动作用。

(二)推动商城走上国际化。围绕建设“三中心、两高地”，以国际化带动临沂商城加快转型。力争临沂商城市场交易额突破4000亿元，全市物流总额、电子商务交易额、进出口总额分别达到2.7万亿元、1500亿元和92亿美元。加快建设“国际商贸城”，完善基础设施，推进电子商务产业园、大数据服务中心、贸易商总部基地等项目，打造高端业态发展区和国际贸易发展样板区。改造提升“老城市场”，优化资源配置，推动传统市场集约化发展。着力打造“网上商城”，在天猫国际等跨境电商平台设立临沂商城海外专区，构建电商全产业链基地，积极争创全国跨境电子商务综合试验区。走出去建设“海外商城”，加强与有海外工程项目的央企合作，布局跨境电商“边境仓”、“海外仓”。提升发展公路物流，依托智慧物流信息平台，发展第三方、第四方物流，加快中心城区保税区国际物流园、高速物流园等项目建设。大力发展铁路物流，新开行临沂至成都、重庆、义乌、福州等地货运班列，形成通达全国、连接“一带一路”的铁路物流网络。加快发展航空物流，完成临沂机场改扩建工程，开行全货机航线，增开国际和地区航线，加快建设国家一类航空口岸;推进航空物流区和国际航空快件监管中心建设，吸引航空物流企业在临沂机场建立国际、国内分拨中心。着力发展临港物流，完善通港基础设施，引进仓储物流项目，打造千亿级港口物流产业。发挥综保区政策优势，建设跨境电子商务公共服务平台，打造商城国际化新窗口。扩大临沂港出口直通范围，争取实现进口直通，早日纳入国际陆港城市序列。继续办好各类展会，争取成为鲁南苏北区域性会展中心城市。

(三)推动城乡统筹发展。坚持规划引领、基础设施先行和产城融合，推进城镇化由外延扩张向内涵提升转变。实施“四级联动”，促进城乡一体发展。中心城区，高标准编制高铁片区、轨道交通、海绵城市、地下综合管廊等专项规划。加快实施12大类176个基础设施和重点工程，优先发展道路桥梁、地下管网、公共交通、水环境治理、园林绿化等项目，提升城市综合承载能力。大力推进鲁南高铁临沂至曲阜段、临沂至日照段和罗岚高速公路建设，开工建设新西外环路，完成双岭高架路，拓宽改造北京东路，加快西安路、临西十一路地下综合管廊建设，新增供水、供热、供气管网30公里、20公里和90公里，实施143个老旧小区改造，建设便民市场14处、公交场站5处。县城，尽快编制县域城市功能区、历史文化街区保护等专项规划，优化空间布局和产业结构。把棚户区改造与推进县城功能提升、环境改善结合起来，强化政策和资金支持，确保全面完成棚改任务。着力抓好园区规划建设，进一步完善基础设施，增强孵化功能，为产业发展搭建载体。小城镇，按照因地制宜、分类指导的原则，重点抓好一批特色鲜明的新市镇和重点镇。引导各乡镇立足产业、生态、文化等优势，大力发展生态农业、乡村旅游和农产品深加工等特色产业。农村，以完善设施配套、改善人居环境为重点，实施城乡环卫一体化、农村汪塘整治和生活污水处理、新型社区建设，加强乡村风情风貌保护，打造一批美丽乡村。新建、改建农村公路500公里，新增镇村公交线路18条、公交车辆100台。坚持问题导向，着力破解城镇化进程中的瓶颈制约。发挥财政资金“四两拨千斤”的作用，积极引入社会资本参与新型城镇化建设。全面放开落户限制，健全居住证与户籍衔接的人口管理机制，稳步实现教育、医疗、住房、社保等基本公共服务常住人口全覆盖，让有能力在城镇稳定就业的外来人口举家进城落户。通过房贷补贴、租购结合、政府购买、棚改货币化安置等办法，进一步释放购房需求，改善住房供给结构。积极推进智慧临沂建设，完善信息基础设施，搭建教育、健康、社区等智慧云平台。巩固全国文明城市、国家卫生城市创建成果，完善常态长效机制，不断提高城乡管理水平。

(四)推动改革释放新动能。突出重点领域和关键环节，加强供给侧结构性改革。深化行政审批制度改革，强化事中事后监管，再取消下放一批行政审批事项，降低制度性交易成本，减轻企业税费负担。深入推进国有企业改革，明晰国有集体企业产权关系，建立法人治理结构，激发企业发展活力。认真落实中央深化税收体制改革要求，按时完成“营改增”任务。调整财政支出结构，大力压减“三公经费”等一般性支出，腾出更多资金用于基本公共服务。合理控制政府债务规模，有效化解政府债务风险。支持企业实行主辅分离，将服务业从主业中剥离，形成独立纳税主体。认真落实金融支农支小和金融扶贫政策，积极稳妥推进民间融资规范管理试点和新型农村合作金融试点。加快农信社银行化改革，上半年全部完成改制任务。加强金融风险监测，妥善处理风险案件，坚决守住不发生系统性和区域性金融风险的底线。严格落实小微企业税收优惠政策，为创业创新减负。

(五)推动环保步入常态化。以建设国家生态文明先行示范区为抓手，守住资源消耗上限、环境质量底线、生态保护红线，争取成为后发地区绿色赶超、科学发展典范。坚决将大气污染防治进行到底，深度治理污染行业，统筹做好能源结构调整、城市扬尘治理、机动车尾气治理、秸秆禁烧等工作，持续改善环境空气质量。提升中心城区水环境，完成青龙河等主要河流的综合整治任务，彻底解决城区河流水质超标问题。严格新建项目能耗达标管理，推进工业、建筑、交通、公共机构等重点领域节能。推行环境污染第三方治理，实行排污权有偿使用和交易，提高环境污染治理市场化、专业化水平。搞好沿路、沿河、沿水库、城郊和山岭造林绿化，市域森林覆盖率提高到35.6%以上。落实最严格的水资源管理制度，加强雨洪资源开发利用，加快建设河湾水库、袁家口子水库等水源工程，完成姜庄湖拦河坝、岸堤水库和石泉湖扩容等工程，缓解水资源供需矛盾。加强矿山地质保护和修复，治理水土流失300平方公里，完成208座塘坝除险加固，打造100座汪塘整治示范工程，促进生态环境持续改善。

(六)推动民生建设落到实处。按照人人参与、人人尽力、人人享有的要求，建设一批民生项目，办好一批民生实事，解决一批民生难题，努力让群众分享改革发展成果。以“一点两区”为示范，统筹抓好产业扶贫、就业扶贫、教育扶贫、健康扶贫、金融扶贫、搬迁扶贫、社保扶贫和社会扶贫，确保年内再减贫26万人。提升农村饮水安全保障能力，新增受益人口20万人。整合劳动力资源市场，打造“云就业”服务平台，促进供需双方高效对接。统筹推进各类教育发展，新建、改扩建城镇学校68所、校舍108万平方米，消化50%左右的“大班额”;规划新建农村校舍19万平方米，改厕、取暖工程覆盖率分别达到100%和50%，完成农村学校年度“全面改薄”任务。深化爱国卫生运动，启动城市公立医院综合改革，加快市人民医院北城新区医院、肿瘤医院新院等项目进度，新增城区医疗卫生机构10处，完成乡镇中心卫生院项目建设，新建村卫生室50个，县域内就诊率提高到90%以上。大力发展城市公共交通，新增公交车500台、网络预约出租车300台，建设BRT快速公交，投运新能源汽车充电站30座，方便群众出行。建立城区公共自行车系统，上半年800个站点、2万辆自行车投入运营。抓好北城新区部分道路改造，增设重点道路非机动车道和行人专用道，建设滨河下穿通道，提高交通安全指数。进一步规范物业管理，城区住宅小区物业覆盖率提高到70%。管好用好各类文化体育设施，开展群众性文化体育活动，办好书圣文化节、沂河国际运动娱乐节等节会赛事，建设蒙山国家登山健身步道。强化食品药品安全源头治理，抓好农贸市场、大型超市、学校食堂等重点领域监管，保障群众饮食用药安全。扎实推进平安临沂建设，健全完善社会治安立体化防控体系，维护社会和谐稳定。全面落实安全生产责任制，突出煤矿和非煤矿山、危化品、商场消防以及交通、建筑等重点领域，集中开展安全生产大检查、大整改、严执法行动，坚决打好安全生产翻身仗，尽快扭转安全生产被动局面。全面落实两孩政策，提升计生服务管理水平，促进人口均衡发展。加强村居干部经济责任审计，搞好村民自治和村务监督。扎实做好双拥共建，推动军民融合发展。抓好防汛抗旱、气象、地震、人防等工作，完善消防安全设施，提高防灾减灾和应急救援能力。大力开展文明创建、道德模范评选、乡村移风易俗等活动，唱响主旋律，树立新风尚。四、关于政府自身建设

各位代表，面对经济发展进入转型期、改革攻坚步入深水区、社会治理转入新阶段的新形势，各级政府必须忠实履行宪法和法律赋予的神圣职责，始终与群众心心相印，夙夜在公、勤勉工作，努力向人民交上一份合格的答卷。

第一，敢于担当不懈怠。在担当中点燃激情、激发干劲，把精力放在狠抓落实上，把心思放在干事创业上，把本领用在促进发展上，创造经得起历史检验的业绩。在担当中克难前行、不辱使命，用改革精神、创新办法和法治思维改进工作，敢于触及深层矛盾，打破传统路径依赖，不断开创工作新局面。在担当中守土有责、守土尽责，健全完善目标责任、执行监督和绩效考核机制，对实绩突出的大力褒奖，对为官不为、懒政怠政的公开曝光、坚决问责，确保有令必行、行必有果。

第二，依法行政严用权。全面推进法治临沂建设，树立权由法定、权依法使的理念，在法治轨道上开展政府工作。自觉运用法治思维和法治方式深化改革、推动发展，努力营造良好法治环境。强化决策法定程序的刚性约束，坚持重大行政决策集体讨论决定，健全公众参与、专家论证、风险评估、合法性审查制度，确保依法、科学、民主决策。自觉接受人大及其常委会监督，定期报告政府工作。主动加强同人民政协的民主协商，大力发展基层协商民主。做好人大代表建议和政协委员提案办理工作。注重发挥法律顾问、律师和司法机关作用。积极推进政府信息公开，自觉接受各方监督。

第三，优化服务提效能。把打造“基层办事无障碍、企业办事无投诉、群众办事无难处”的优良环境作为目标，健全基本公共服务体系，提高政务服务事项和市民服务热线办理实效，及时查处行政不作为、乱作为、慢作为行为。严格落实领导干部联系和服务群众制度，深化“结亲连心”活动，推动机关干部走入基层、服务群众。认真履行向社会作出的各项承诺，把好事办好、实事办实，用干部的辛苦指数提升群众的幸福指数。

第四，清正廉洁树形象。严明政治纪律，严守政治规矩，落实党风廉政建设责任制，坚决抵制“舌尖上”的腐败，杜绝“车轮上”的铺张，避免“钱袋子”的浪费，持续巩固党的群众路线教育实践活动和“三严三实”专题教育成果。坚持用制度管权、管事、管人，健全惩治和预防腐败体系，完善防范权力寻租和利益输送的体制机制，切实把权力关进制度的笼子。强化行政监察，推进审计监督全覆盖，严肃查处各类违纪违法案件，坚决纠正损害群众利益的不正之风，始终保持清廉本色，做群众满意公仆。

各位代表，新征程任重道远，新目标催人奋进。让我们在市委的坚强领导下，扬改革创新之帆、践真抓实干之行、求惠民利民之效，凝心聚力，团结拼搏，为加快建设社会主义“大美新”临沂作出新的更大贡献!
